# Amyloidogenic Properties of a D/N Mutated 12 Amino Acid Fragment of the *C*-Terminal Domain of the Cholesteryl-Ester Transfer Protein (CETP)

**DOI:** 10.3390/ijms12032019

**Published:** 2011-03-21

**Authors:** Victor García-González, Jaime Mas-Oliva

**Affiliations:** Institute of Cell Physiology, National Autonomus University of Mexico (UNAM), AP 70-243, 04510 Mexico, D.F., Mexico; E-Mail: vgarcia@emailifc.unam.mx

**Keywords:** cholesteryl-ester transfer protein (CETP), CETP C-terminal domain, α-helix and β-sheet secondary structures, peptide oligomers, amyloids

## Abstract

The cholesteryl-ester transfer protein (CETP) facilitates the transfer of cholesterol esters and triglycerides between lipoproteins in plasma where the critical site for its function is situated in the *C*-terminal domain. Our group has previously shown that this domain presents conformational changes in a non-lipid environment when the mutation D_470_N is introduced. Using a series of peptides derived from this *C*-terminal domain, the present study shows that these changes favor the induction of a secondary β-structure as characterized by spectroscopic analysis and fluorescence techniques. From this type of secondary structure, the formation of peptide aggregates and fibrillar structures with amyloid characteristics induced cytotoxicity in microglial cells in culture. These supramolecular structures promote cell cytotoxicity through the formation of reactive oxygen species (ROS) and change the balance of a series of proteins that control the process of endocytosis, similar to that observed when β-amyloid fibrils are employed. Therefore, a fine balance between the highly dynamic secondary structure of the *C*-terminal domain of CETP, the net charge, and the physicochemical characteristics of the surrounding microenvironment define the type of secondary structure acquired. Changes in this balance might favor misfolding in this region, which would alter the lipid transfer capacity conducted by CETP, favoring its propensity to substitute its physiological function.

## 1. Introduction

To date, a significant number of diseases caused by the aggregation of misfolded proteins have been described. Of these, approximately 40 diseases present amyloid properties associated with the extracellular and intracellular deposition of peptides and proteins [1]. However, self-assembly into fibrillar structures is not a feature restricted to a small group of peptides and proteins with specific patterns in their amino acid sequence or three-dimensional structure, since in the past ten years several peptide sequences have been found not to be related to disease despite their ability to form amyloid structures. The adaptation of specific cellular processes coupled to the formation of amyloids has also been found; for instance, during the polymerization of melanin precursor molecules in melanocytes [2]. Therefore, nowadays the formation of amyloid structures has been considered a property that may be generic to many polypeptide chains and in many cases directly related to function [3].

For several years, our laboratory has been interested in the study of the relationship between structure and function of proteins involved in lipid binding and transport. Secondary to changes found in the secondary structure of specific regions of several of these proteins such as apolipoprotein C–I, we have proposed the possibility that these regions respond to specific changes in the microenvironment that surrounds them, and through specific disorder-to-order transitions these changes act as molecular switches that trigger function [4–6].

**Figure 1.**
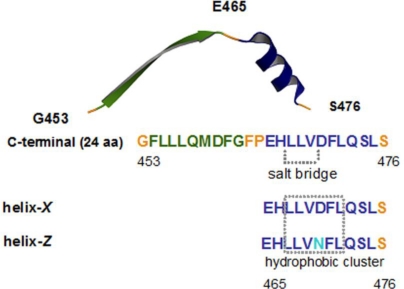
Structural representation of the *C*-terminal region of cholesteryl-ester transfer protein (CETP), showing the sequences of helix-*X* and helix-*Z*. The H_466_–D_470_ salt bridge and the hydrophobic cluster are shown. The structure was obtained from the Protein Data Bank, access code: 2obd.

The cholesteryl-ester transfer protein (CETP) promotes the transfer of cholesterol esters and triglycerides between lipoproteins, mainly directing the cholesterol flow from high density lipoprotein (HDL) to low and very low density lipoproteins (LDL and VLDL). In this regard, extensive studies on genetic polymorphisms and CETP deficiencies suggest a direct relationship between its activity and cardiovascular disease [7]. Site specific mutagenesis studies have shown that the domain located in the *C*-terminus (E_465_–S_476_), structured as an amphipathic α-helix, corresponds to a key region in lipid transfer (Figure 1) [8–10]. During the structural characterization of this domain, we have found that disruption of the H_466_–D_470_ salt bridge across the D_470_N mutation causes the loss of native structure, due in part to the change of the negatively charged carboxylate group of D for the amide group in N (Figure 1) [8].

Three-dimensional structure resolution has provided a solid basis for the proposal that CETP operates through a carrier mechanism of lipid transfer [11]. Nevertheless, studies from our laboratory suggest that the lipid transfer process can be also directly related to the formation of a lipid micellar system where conservation of the α-helical structure of this region is critical for the process [12]. Therefore, this work focused on the 12 residues of the *C*-terminal domain of CETP, and the structure and function of two peptides derived from this site, the native sequence named helix-*X* and a peptide with the mutation D_470_N appointed helix-*Z* (Figure 1), were characterized. Employing helix-*Z* as an example of hydrogen bonding disturbance has allowed us to demonstrate a conformational change of this region from an α-helix to a β-sheet. Ongoing experiments employing CETPI, an isoform of CETP that lacks the normal C-terminal domain and instead presents a new carboxy-end mainly structured as a β-sheet [13], supports the fact that small order-to-disorder and/or disorder-to-order transitions supported by changes in hydrogen bonding might give proteins an entirely new physiological function [14]. Secondary to the formation of β-sheet structures, helix-*Z—*the D/N mutated 12 amino acid (aa) fragment of the C-terminal domain of CETP*—*shows the consequent formation of oligomers and amyloid-like fibril structures that in turn cause cytotoxic effects similar to those exercised by the β-amyloid peptide.

## 2. Results

### 2.1. Conformational Changes in the *C*-Terminal Domain of CETP

Although previous studies performed by our group have demonstrated the key role of the *C*-terminal domain of CETP during the process of lipid transfer when holding an α-helix conformation (residues E_465_–S_476_) [8,13], it has not been determined yet whether this domain presents the ability to undergo conformational changes. Therefore, in order to investigate if this domain might undergo conformational changes by modifying the physicochemical properties of the surrounding media, two peptides were synthesized: the native *C*-terminal peptide called helix-*X*, and a second one containing the mutation D_470_N and named helix-*Z* (Figure 1).

**Figure 2.**
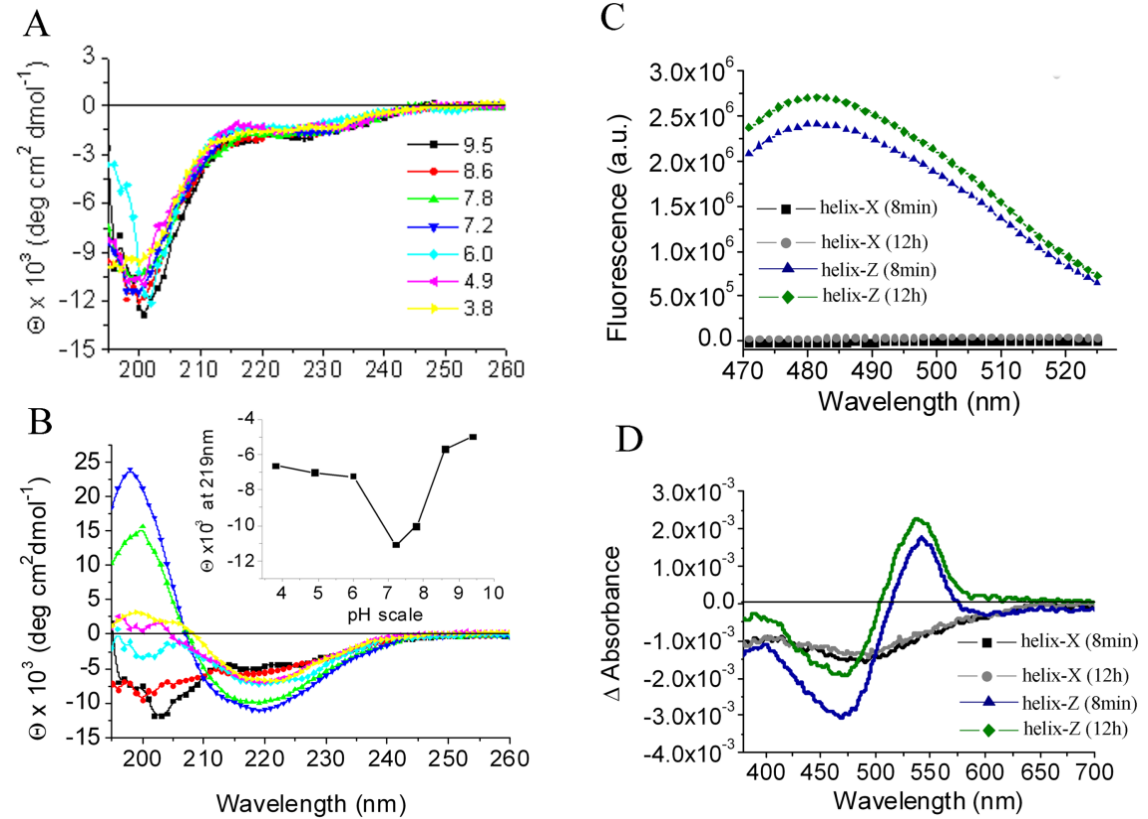
Circular dichroism (CD) spectra of helix-*X* (**A**) and helix-*Z* (**B**) in a pH range of 3.8–9.5. Inset in (**B**) shows ellipticity values at 219 nm; (**C**) Fluorescence assay coupled to ThT, showing a characteristic peak at 482 nm; (**D**) Birefringence changes of Congo red (Δ absorbance) at different incubation times. The peptide concentration used in all CD assays was 200 μg/mL, for ThT assays was 50 μg/mL, and for Congo red assays 180 μg/mL.

Helix-*X* and helix-*Z* were incubated in water in a pH range of 3.8–9.5 and studied by circular dichroism (CD) spectroscopy, in parallel. The secondary structure of helix-*X* was consistent with the presence of a disordered state over the whole range of pH values tested (Figure 2A). In contrast, CD spectra of helix-*Z* showed a pH specific response with minimum shift signals from 219 nm to 201 nm, which is associated with an increase in ellipticity indicative of the formation of β-type secondary structures (Figure 2B). This spectrum was only found in a pH range of 7–8, reaching the highest value at pH 7.2 (Figure 2B insert).

Moreover, through the use of thioflavin-T (ThT) fluorescence assays, an increase in the emission spectrum of helix-*Z* with a maximum signal near 482 nm was observed (Figure 2C). Also, by testing with a Congo red-shift assay, the characteristic birefringence change observed in the presence of β-structures with a maximum signal close to 540 nm was determined (Figure 2D). Therefore, these assays confirm the possibility that under specific conditions the *C*-terminal domain of CETP can acquire a secondary structure consistent with the formation of a β-structure.

**Figure 3.**
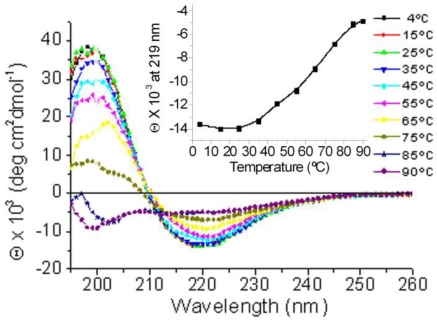
Temperature-induced unfolding of helix-*Z*. CD spectra were obtained from 4 to 90 °C. Inset shows ellipticity values at 219 nm (β-structure signal). The concentration of helix-*Z* employed was 200 μg/mL.

During the evaluation of the stability of helix-*Z* through temperature-induced unfolding CD assays (4–90 °C), only temperatures close to 65 °C were found to induce significant loss in its β-type secondary structure, suggesting high stability. The presence of an isodichroic point near 211 nm in the obtained spectra is associated with the presence of only two conformational states (Figure 3). These experiments, together with the fact that helix-*Z* keeps its characteristic CD profile consistent with a β-strand under a range of high ionic strength solutions (up to 2.4 M NaCl, data not shown), show that the acquired β-structure is not determined by long-range electrostatic interactions, but rather by stronger inter- and intra-chain interactions such as hydrogen bonds.

**Figure 4.**
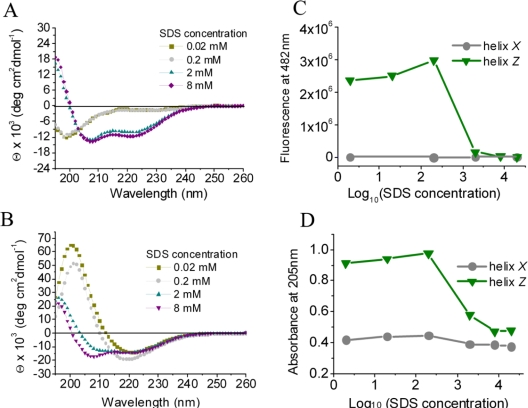
The effect of SDS on the secondary structure of peptides monitored by CD. (**A**) helix-*X*; (**B**) Helix-*Z*; (**C**) ThT fluorescence at 482 nm; (**D**) Absorbance at 205 nm. The peptide concentration used in all CD assays was 200 μg/mL. For ThT assays was 50 μg/mL and for absorbance assays 180 μg/mL.

Another set of experiments, where both helix-*X* and helix-*Z* were incubated in a hydrophobic environment composed of sodium dodecyl sulfate (SDS) micelles, shows that both peptides acquire and maintain an α-helical conformation (Figure 4A,B). This transition was monitored in parallel following fluorescence coupled to ThT (Figure 4C) and the change in absorbance of peptide bonds at 205 nm (Figure 4D). Our results suggest that the interaction within a hydrophilic/hydrophobic interface may be the key parameter to maintain both peptides in an α-helix conformation; more importantly, in the case of helix-*Z*, this is a key feature to avoid the formation of a β-structure.

### 2.2. D_470_N Mutation in the *C*-Terminal Domain of CETP Induces Amyloid Fibril Formation

**Figure 5.**
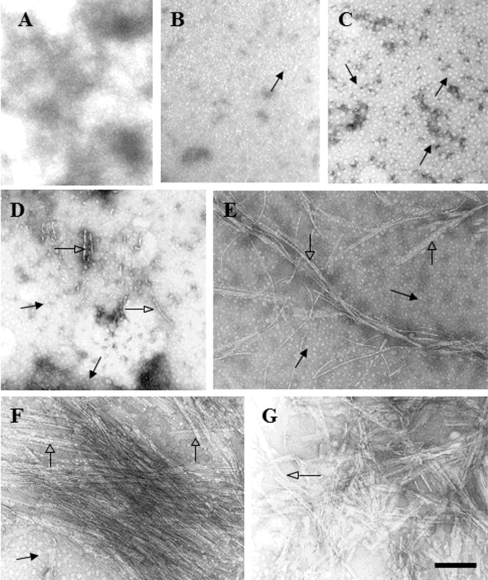
Ultrastructural analysis of peptides by transmission electron microscopy (TEM); (**A**) Helix-*X* at 48 h of incubation at 37°C; (**B**) Helix-*Z* at 0 h of incubation; (**C**) Helix-*Z* at 0.5 h of incubation; (**D**) Formation of helix-*Z* protofilaments at 1.0 h of incubation; (**E**) Helix-*Z* under constant agitation at 6.0 h of incubation; (**F**) Helix-*Z* under constant agitation and 48 h of incubation; (**G**) Helix-*Z* under constant agitation at 120 h of incubation. Solid arrows indicate oligomeric structures, open arrows show protofibrils and amyloid fibrils. The concentration of peptide used in all conditions was 60 μg/mL. Bar indicates 200 nm.

In order to extend our structural analysis and considering that an increase in β-signal is not a direct indication of the formation of amyloid fibrils, peptide samples were analyzed by transmission electron microscopy (TEM). Throughout the pH-range evaluated (4–9), helix-*X* samples did not form any kind of structured material, which correlates well with previous results in which helix-*X* remains in a non-ordered state (Figure 5A). In contrast, when helix-*Z* samples were analyzed, even from the first sample without incubation (time 0), small oligomers were clearly identified (Figure 5B,C). It was only after 1 h of incubation that protofilament formation was observed (Figure 5D).

Incubation of helix-*Z* for 6 h with stirring (200 rpm) was the condition that identified a mixture of oligomeric structures, protofilaments, and mature fibrils in some areas (Figure 5E). Samples analyzed after an incubation time of 72 and 120 h at 37 °C clearly showed mature fibrils with high morphological similarities to β-amyloid samples (Figure 5F,G). At this stage, it is interesting to mention that although the formation of fibrils from pH 4–9 was evaluated, aggregates and fibril formation were only observed at pH 7–8, which correlates well with the presence of β-structures at this pH range when monitored by spectrophotometric techniques.

### 2.3. Cytotoxic Effects Associated with Helix-*Z*

**Figure 6.**
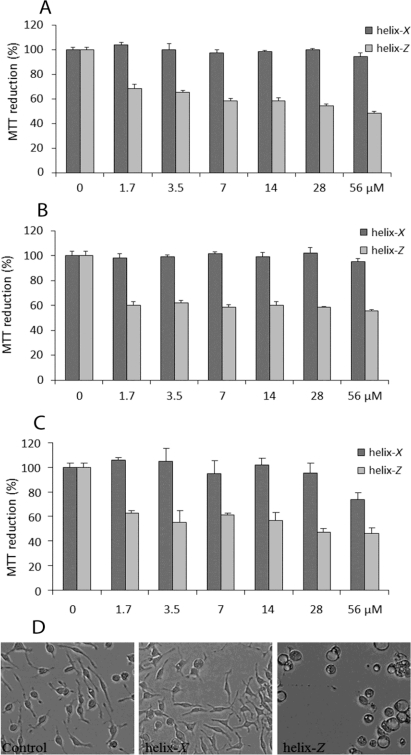
Cytotoxic effects in microglia associated with the exposure of helix-*X* and helix-*Z*. (**A**) Cell viability determined by MTT for cells treated with peptide previously incubated 0.5 h at 37 °C; (**B**) Cells treated with peptide previously incubated 6 h at 37 °C; (**C**) Cells treated with peptide previously incubated 120 h at 37 °C; (**D**) Light microscopy showing microglia treated with both peptides (56 μM) previously incubated 120 h at 37 °C.

Taking into account previous studies from several laboratories where microglia and macrophages were employed in the characterization of the cytotoxic effects of the β-amyloid peptide [15–17], when microglial cells were exposed to a gradual increase in concentrations of both peptides, only helix-*Z* was able to significantly reduce cell viability as measured by the 3-(4,5-dimethylthiazol-2-yl)-2,5-diphenyltetrazolium bromide (MTT) reduction assay (Figure 6). It is interesting to observe that independently of the incubation time used for both peptides in solution (0, 6, 120 h) the lowest concentration employed for helix-*Z* (1.7 μM) shows an important reduction in cell viability that is maintained up to 56 μM (Figure 6A–C). At this stage it is important to mention that 0 time actually corresponds to the time employed to place both peptides, first in solution and second into the cell culture plate. This procedure takes approximately 15 min, enough time to apparently produce peptide oligomers as shown in Figure 5B–C. Because cell viability is maintained close to 50% independently of the concentration of helix-*Z* or the time of the incubation needed for the formation of amyloid structures, our experiments indicate that helix-*Z* in the form of oligomers seem to be the highest toxic form for this peptide. Whether or not mature fibrils are also responsible for cell toxicity after the incubation of helix-*Z* for 120 h, will have to be elucidated in experiments where under these conditions the presence of small amounts of oligomers can be ruled out. At this stage it is interesting to mention that while helix-*Z* oligomers formed at pH 7.2 are cytotoxic, amorphous aggregates of the β-amyloid peptide obtained at the same pH do not present the ability to damage microglial cells in culture (Figure 1 supplementary data). Changes found in cell morphology are possibly related to the presence of a cellular stress condition as described by our group when studying the β-amyloid peptide [17] (Figure 6D). It is noteworthy that the MTT assay is also an indicator of oxidative stress and changes in the vesicular trafficking of cells [18,19].

### 2.4. Endocytic Protein Expression

**Figure 7.**
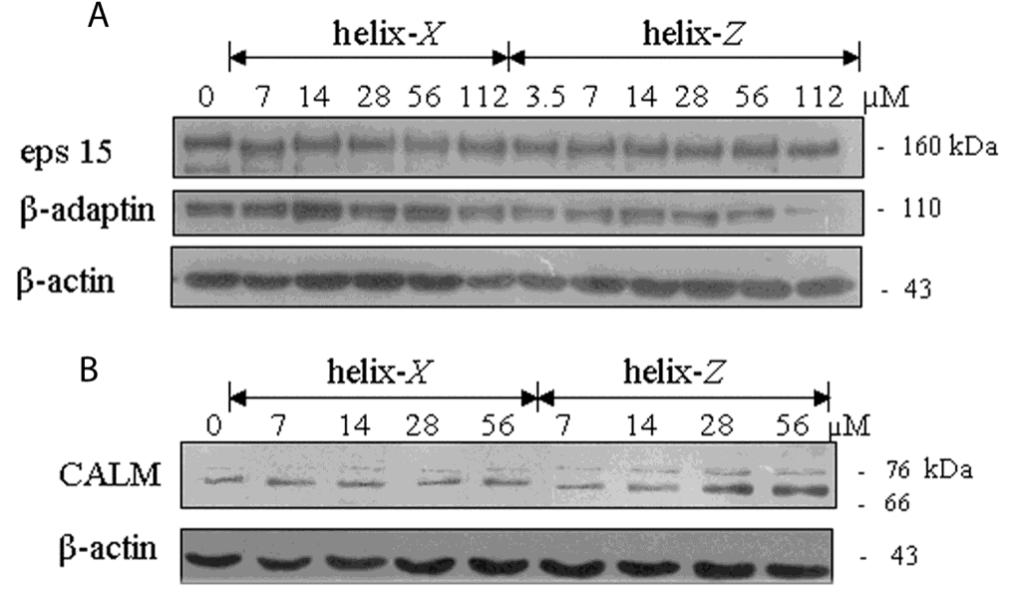
Endocytic protein expression in microglial cells treated with helix-*X* and helix-*Z*. Both peptides were previously incubated 120 h at 37 °C. (**A**) Western blot analysis of eps 15 and β–adaptin; (**B**) Western blot analysis of clathrin assembly lymphoid myeloid leukemia (CALM) protein. β-actin was used as loading control.

After an incubation period of 120 h at 37 °C (condition at which mature fibrils are obtained with helix-*Z*) microglial cells were treated for 20 h with increasing concentrations of peptides. The experiment was focused on assessing the expression levels of proteins such as β-adaptin, eps 15, and clathrin assembly lymphoid myeloid leukemia (CALM). Cells exposed to helix-*Z* showed differential expression of some of these endocytic proteins, in contrast to experiments performed with helix-*X* in which no changes were found. While the expression of proteins such as eps 15 was held constant, β-adaptin protein expression decreased with respect to exposure to increasing concentrations of helix-*Z* (Figure 7A). In contrast, CALM protein expression increased with respect to the concentration of helix-*Z* (Figure 7B). These results, as shown previously by us, correlate well with the β-amyloid peptide (Aβ_25–35_) experiments that were employed under the same conditions as used here with helix-*Z* (Figure 1 supplementary data).

## 3. Discussion

During the search for factors that might explain the formation of a β-structure in helix-*Z*, we propose that this structural feature is not determined by parameters such as the hydrophobic moment (μH) or the mean hydrophobicity, since differences in values with respect to helix-*X* are minimal (Table 1). However, we found that helix-*Z* presents a β-sheet structure, only when it presents a net charge close to −1 in the pH range of 7–8. In contrast, helix-*X* with a net charge closer to −2 remains in a non-structured state (Table 2).

In this sense, there are reports that show a higher propensity to form β-structures when a low net charge and a high hydrophobicity are present in the peptide sequence, conditions that promote amyloid fibril formation [20–22]. Considering these factors, Lopez de la Paz *et al.* [23] have reported that peptide sequences are capable of forming fibrils only when they present a net charge of +1 or −1 and distances between charges are maximized, allowing fibrillogenesis in an orderly form as observed with region 25–35 of the β-amyloid peptide (Table 2). This phenomenon has been proposed as a key feature in molecular evolution preventing the occurrence of misfolding phenomena [24]. Also, there is strong evidence that intrinsically disordered proteins maintain a high net charge as a strategy to prevent aggregation [25]. Therefore, net charge may be the factor that triggers the orderly fibril formation shown by helix-*Z.* It is considered to be a two-stage process with a lag period related to the formation of aggregates and nucleation centers and a second stage related to fast spreading [26].

**Figure 8.**
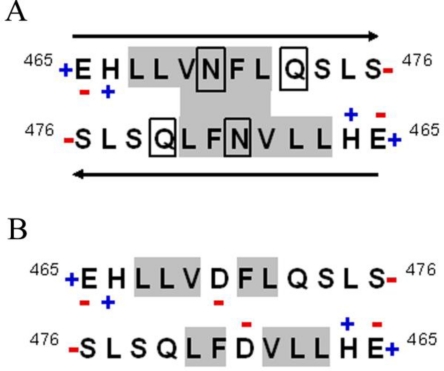
Model of an antiparallel β-strand in helix-*Z*. (**A**) The charge of each residue is determined considering a pH of 7.2. *N* and *C* terminal groups are loaded allowing an electrostatic attraction between peptide chains. Shaded regions identify the hydrophobic cluster L_467_LVNFL_472_, as well as N and Q residues; (**B**) Helix-*X* does not form a β-strand structure due to the electrostatic repulsion between D residues.

Taking into account the spectrophotometric results, the evidence of fibrillar structures and the physicochemical properties of helix-*Z* such as μH, hydrophobicity, and a net charge of −0.96 at pH 7.2, we present a model for the amino acid residue disposition within the β-strand (Figure 8A). Since D_470_N showed the loss of one negative charge, the hydrophobic cluster (L_467_LVNFL_472_) maintained a β-conformation through hydrophobic interactions. It is also important to consider that along the sequence, there are asparagine and glutamine residues that are considered as inducers of β-structures and therefore allow the stacking of polypeptide chains [27]. Helix-*X* does not show this phenomenon, largely due to an electrostatic repulsion at Asp-470 that prevents the formation of a hydrophobic cluster that maintains the peptide under an extended state (Figure 8B).

On the other hand, it is surprising that helix-*X* is maintained as an α-helix in the crystallographic data shown for CETP due to its μH value of 0.41 kcal/mol, factor B value, and relative freedom from the main body of the protein. In this regard, peptide sequences showing the potential for intrinsic disorder and domains with high factor-B values are associated with a high thermal vibration of individual atoms and therefore present high intramolecular flexibility [28]. Although helix-*X* presents an α-helix profile in the crystal structure of CETP, when held outside a lipid environment it might adopt a disordered structure with the possibility that this region might undergo disorder-to-order and/or order-to-disorder transitions. This type of transition must probably present a different equilibrium when changes like the one found in helix-*Z* is introduced to this region since the formation of a β-sheet might not be reversible.

Oil-water interfaces also present the ability to promote the stabilization of the secondary structure of peptides believed to the due to hydrogen bonding and a reduction of the energetic cost of partitioning [29]. As previously suggested for the bee venom peptide mellitin, this peptide is largely unstructured when placed in solution, but highly structured as an amphipathic α-helix when partitioned into unilamellar phospholipid vesicles [30]. While the free energy reduction per residue observed for the folding of mellitin in a membrane interface is almost 0.4 kcal mol^−1^ consistent with the formation of hydrogen bonding, values close to 0.6 kcal mol^−1^ have been also shown for β-sheet forming peptides [30].

Studies performed by our group using β-amyloid fibrils as a natural ligand for the scavenger receptor (SR), an association directly related to the development of oxidative stress, have shown changes in the expression of several adaptor proteins involved in the endocytic machinery of macrophages and microglia [17,31]. Proteins α-, β-, μ-, and σ-adaptins integrate into the AP2 adaptor complex, a key component in the initial stages of endocytosis [5]. Within the mechanism of clathrin-mediated endocytosis of the SR, more than 30 proteins have been described, many of which are adaptor molecules involved in the formation of coated pits. In addition to clathrin, proteins such as CALM, epsin, dynamin, amphiphysin, and eps 15 also play an important role in this process [32].

The present results show that when microglial cells are exposed to helix-*Z*, peptide oligomers first and fibrillar species next, are the leading cause for the registered cytotoxic phenomena. Moreover, high concentrations of helix-*Z* fibrils inhibit β-adaptin expression. With the decline of β-adaptin, the increased expression observed for CALM could function as a cellular compensatory mechanism for endocytic function. In this sense, the ANTH domain (AP180 N-Terminal Homology) located in the first 300 residues of CALM allows interaction with PtdIns(4,5)P_2_ [33], functioning as a bridge between the plasma membrane clathrin-coated pit and other adaptor proteins [34]. Taking into account that a similar response in β-adaptin and CALM was found in this study when cells were exposed to helix-*Z* fibrils, possibly this condition may be associated with a phenomenon found in cells that come in contact with toxic peptide oligomers and/or amyloid fibrils.

## 4. Experimental Section

### 4.1. Materials

All cell culture reagents were purchased from Gibco-Invitrogen (Carlsbard, CA, USA), while tissue culture dishes and other plasticware were obtained from Nulgene Nunc (Rochester, NY, USA). Salts and buffers were purchased from Baker. Tert-butyl hydroperoxide (TBH), ThT, Congo-red, SDS and MTT were obtained from Sigma (St. Louis, MO, USA). Antibodies used in CALM, β-adaptin, and eps 15 protein detection as well as goat secondary antibodies were from Santa Cruz Biotechnoloy Inc.

### 4.2. Peptide Synthesis and Preparation

Based on the function of the *C*-terminal of CETP, two peptides were synthesized: one named helix-*X* that corresponds to the native peptide, and the second one named helix-*Z* containing the mutation D_470_N. Lyophilized peptides were dissolved in ammonium carbonate buffer (pH 9.5) to a concentration of 1 mg/mL. From this solution a further 1:5 dilution was carried out. To evaluate the structure at pH 3.8 and 4.8, a sodium acetate buffer was used; pH 6.3 and 7.2, a sodium phosphate buffer was used, and at pH 8.6 and 9.5, an ammonium carbonate buffer was employed.

Fragment Aβ_25–35_ (β-amyloid) was employed as a reference molecule for the cytotoxicity assays. Experiments performed with β-amyloid were carried out at two pH conditions using a phosphate buffer (pH 7.2) and ultrapure water (pH 5.5), both with the same peptide concentration (1.5 mg/mL). All buffers were used at 50 mM, and solutions were filtered through 0.22 μm membrane filters before use.

Purity of peptides was greater than 98% (GenScript Corp., Piscataway, NJ, USA). Their identity and purity were confirmed by mass spectrometry and HPLC analysis (data not shown). Protein concentration was determined by peptide bond absorption at 205 nm.

### 4.3. Circular Dichroism Spectroscopy

Far-UV CD spectra were recorded on an Aviv 62DS spectropolarimeter in a 0.1 cm quartz cell, using an average time of 2.5 s and a step size of 0.5 nm. CD results are reported as mean molar ellipticity (Θ, deg cm^2^ dmol^−1^) considering the baseline correction. For the pH studies, CD spectra were obtained from pH 3.8 to −9.5. Peptide unfolding induced by temperature was monitored by CD measurements over a temperature range of 4 to 90 °C.

### 4.4. Congo Red Spectroscopy and Thioflavin T Fluorescence

Congo red assays were performed based in the protocol designed by Klunk *et al.* [35]. Employing 10.6 μM Congo red and 180 μg/mL of peptide, the absorbance was measured every 2 nm using a Perkin Elmer UV/Vis Lambda 2*S* spectrophotometer scanning from 360 to −700 nm. Peptide spectra were corrected by subtracting the corresponding buffer spectra obtained under identical conditions. Additionally, the presence of β-structures was characterized with the ThT assay. ThT fluorescence emission spectra were registered at 37 °C from 470 to 530 nm with an excitation wavelength of 450 nm. A scan velocity of 73 nm/min using an Olis DM45 spectrofluorimeter was used. The concentrations of ThT and peptides were 10 μM and 36 μM, respectively.

### 4.5. Cell Culture

EOC cells (mouse microglia, American Type Culture Collection) were grown in Dulbecco’s modified Eagle’s medium (DMEM) supplemented with 10% fetal bovine serum, and 20% mouse bone marrow, producing colony stimulating factor-1 (LADMAC) conditioned media. Penicillin (50 U/mL) and streptomycin (50 μg/mL) were added to the media. After the initial culture, all experiments were performed using Opti-MEM medium without phenol red and reduced fetal serum.

### 4.6. Cell Viability Assay

Peptide cytotoxicity was assessed by MTT reduction assays in EOC cells exposed to helix-*X*, helix-*Z*, and the β-amyloid peptide (Aβ_25–35_). Cells were seeded into 96-well plates at a density of 1 × 10^4^ cells/well and allowed to grow to 80% confluence. Next, culture medium was replaced with Opti-MEM medium. After 2 h under this condition, cells were treated with a series of gradually increasing peptide concentrations for 20 h. After this time, 30 μL of an MTT stock solution in Opti-MEM medium was added to the cultures in order to obtain a final concentration of 0.5 mg/mL. Formazan crystals that formed after 4 h of incubation were further dissolved by the addition of cell lysis buffer (20% SDS, 50% *N*,*N*-dimethylformamide, pH 3.7). After 12 h of incubation, absorbance was measured at 570 nm using a microplate reader.

### 4.7. Western Blot Analysis

With a plate confluence of 80%, cells were treated for 20 h with different doses of peptides. After this procedure, cells were washed with PBS and lysed for 45 min at 4 °C in lysis buffer. Lysed cells were centrifuged at 5000 rpm for 5 min and the supernatant was recovered. Protein concentration was determined with the BCA protein assay (Pierce, Rockford IL, USA) and samples (14 μg/lane) from the total protein fraction were analyzed by SDS-PAGE on 8% gels and further transferred to PVDF membranes (Millipore, Billerica, MA, USA). Membranes were blocked overnight at 4 °C in a solution containing Tris-buffered saline (TBS), 1% tween-20, and 5% non-fat milk. For protein detection, the following primary polyclonal antibodies were used: goat anti-β-adaptin (1:600), CALM (1:600), and rabbit anti-eps 15 (1:2500). In addition, the mouse monoclonal anti β-actin (1:500) was used. The blocked membranes were incubated with the primary antibodies for 1 h a 37 °C. After washing, horseradish peroxidase (HRP) conjugated secondary antibodies (1:5000) were added to the membrane and were incubated for 1h at 37 °C in blocking buffer. The secondary antibodies used were: donkey anti-goat IgG for β-adaptin and CALM, goat anti-rabbit IgG for eps 15, and goat anti-mouse for β-actin. Later, membranes were washed with TBS/1% tween and HRP activity was detected with the Immobilon Western kit (Millipore).

### 4.8. Optical Microscopy

Under the different exposures using variable concentrations of helix-*X*, helix-*Z*, and the β-amyloid peptide (Aβ_25–35_), EOC cells were visualized and images were taken with an Olympus IX71 microscope (100 and 400×).

### 4.9. Electron Microscopy

Peptide samples incubated under the different conditions tested were processed using a negative staining technique and visualized using TEM. Samples (10 μL) stained with uranyl acetate solution (2% w/v) were placed on carbon-coated copper grids (400 mesh); and after incubation for 5 min at 25 °C they were dried for 10 min. TEM images were acquired using a JEM-1200EX11 JEOL microscope (70 kV).

## 5. Conclusions

Since the mutation D_470_N in the *C*-terminal domain of CETP exemplifies the delicate balance that exists in the conformational changes at the secondary structure level modulated by pH and amino acid-charge modification, disruption of this dynamic equilibrium could lead to the formation of new interactions like hydrogen bonds within the hydrocarbon backbone and between side chains, which promote aggregation and the formation of fibril structures [36]. Although the D_470_N mutation (helix-*Z*) described in this investigation has not been found in nature, helix-*X*, the natural 12 aa segment of the *C*-terminal domain of CETP, when partitioned into water or into oil-water interfaces might present potential disorder-to-order and/or order-to-disorder transitions that normally are difficult to identify. Therefore, we have used helix-*Z* in order to mimic potential changes in secondary structure of helix-*X* by simply modifying hydrogen bonding that in turn might explain changes in the overall function of native CETP when studied *in vivo*.

## Figures and Tables

**Table 1.** Physicochemical parameters of peptides.

**Parameter**	**helix-*X***	**helix-*Z***	**Aβ_1–42_**	**Aβ_25–35_**
MW (Da)	1399.8	1399.6	4514	1060.3
Isoelectric point	4.17	5.13	5.21	8.75
Hydrophobicity (kcal/mol)	0.27	0.28	0.21	0.37
μH (kcal/mol)	0.41	0.41	0.08	0.03

**Table 2.** Net charge distribution of peptides as a function of pH.

**pH**	**helix-*X***	**helix-*Z***	**Aβ_1–42_**	**Aβ_25–35_**
4	0.07	0.65	3.17	1.02
4.5	−0.47	0.34	1.55	1.01
5	−0.87	0.06	0.38	1.00
5.5	−1.16	−0.18	−0.49	1.00
6	−1.47	−0.48	−1.42	0.99
6.5	−1.75	−0.75	−2.25	0.99
7	−1.91	−0.91	−2.72	0.99
7.5	−1.97	−0.97	−2.92	0.99
8	−2.00	−1.01	−3.01	0.97
8.5	−2.06	−1.06	−3.1	0.93
9	−2.16	−1.17	−3.32	0.79
9.5	−2.39	−1.39	−3.81	0.52

## Supplementary Data

Figure 1S. Effect of pH on Aβ_25–35_ fibril formation. (**A**) Aβ_25–35_ incubated for 72 h at pH 7.2 induces the formation of amorphous aggregates; (**B**) Microglial cells treated with these amorphous aggregates did not produce changes in endocytic protein expression; (**C**) Incubation of Aβ_25–35_ for 72 h at pH 5.5 induces the formation of well-defined fibrils; (**D**) Cells treated with Aβ_25–35_ fibrils show changes in β-adaptin and CALM expression; (**E**) Well-defined fibrils of Aβ_25–35_ induce a gradual decrease in microglial cell viability. TEM images were obtained with a peptide concentration of 60 μg/mL. Bars correspond to 200 nm.
