# Supplementary figures and images for: Amyloidogenic Properties of a D/N Mutated 12 Amino Acid Fragment of the C-Terminal Domain of the Cholesteryl-Ester Transfer Protein (CETP)
Source: Int J Mol Sci. 2011 Mar 21;12(3):2019–35. doi: 10.3390/ijms12032019 (PMC3111648; doi:10.3390/ijms12032019)

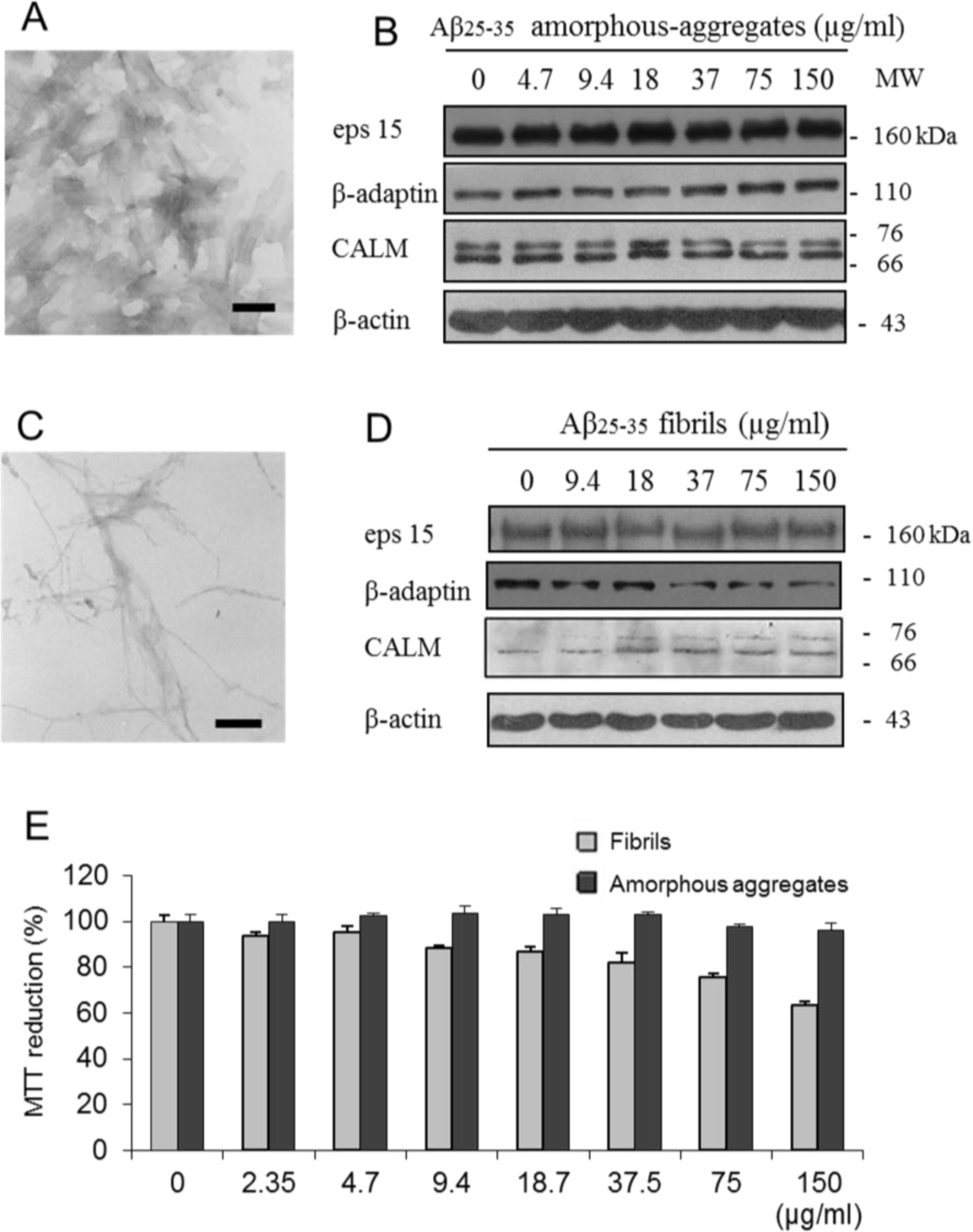

Supplement: Figure 1S. — Effect of pH on Aβ25–35 fibril formation. (A) Aβ25–35 incubated for 72 h at pH 7.2 induces the formation of amorphous aggregates; (B) Microglial cells treated with these amorphous aggregates did not produce changes in endocytic protein expression; (C) Incubation of Aβ25–35 for 72 h at pH 5.5 induces the formation of well-defined fibrils; (D) Cells treated with Aβ25–35 fibrils show changes in β-adaptin and CALM expression; (E) Well-defined fibrils of Aβ25–35 induce a gradual decrease in microglial cell viability. TEM images were obtained with a peptide concentration of 60 μg/mL. Bars correspond to 200 nm. [file ijms-12-02019f9.tif]
